# GPR119 and GPR55 as Receptors for Fatty Acid Ethanolamides, Oleoylethanolamide and Palmitoylethanolamide

**DOI:** 10.3390/ijms22031034

**Published:** 2021-01-21

**Authors:** Dong-Soon Im

**Affiliations:** 1 Laboratory of Pharmacology, College of Pharmacy, Kyung Hee University, Kyungheedae-ro, Dongdaemun-gu, Seoul 02447, Korea; imds@khu.ac.kr; Tel.: +82-2-961-9377; Fax: +82-2-961-9580; 2 Department of Biomedical and Pharmaceutical Sciences, Graduate School, Kyung Hee University, Kyungheedae-ro, Dongdaemun-gu, Seoul 02447, Korea

**Keywords:** GPR119, GPR55, fatty acid ethanolamide, acylethanolamide, oleoylethanolamide, palmitoylethanolamide, arachidonylethanolamide, receptor, G protein-ccoupled receptor

## Abstract

Oleoylethanolamide and palmitoylethanolamide are members of the fatty acid ethanolamide family, also known as acylethanolamides. Their physiological effects, including glucose homeostasis, anti-inflammation, anti-anaphylactic, analgesia, and hypophagia, have been reported. They have affinity for different receptor proteins, including nuclear receptors such as PPARα, channels such as TRPV1, and membrane receptors such as GPR119 and GPR55. In the present review, the pathophysiological functions of fatty acid ethanolamides have been discussed from the perspective of receptor pharmacology and drug discovery.

## 1. Introduction

Arachidonylethanolamide (AEA, also known as anandamide), oleoylethanolamide (OEA), and palmitoylethanolamide (PEA) are members of the acylethanolamide or fatty acid ethanolamide family, and are each composed of a fatty acid and an ethanolamine linked together by an amide bond [1]. These acylethanolamides are present in both animals and plants [2,3]. In particular, the biosynthesis, degradation, and pharmacological actions of acylethanolamides have been studied [4].

Although all acylethanolamides have a common ethanolamide structure, they differ with respect to the attached fatty acid. Therefore, specific receptor proteins recognize them by their differences in the chain length and degree of unsaturation of the fatty acids, which act as agonists/antagonists or activators/blockers. AEA has been extensively studied, and has been identified as an endogenous ligand of cannabinoid CB_1_ and CB_2_ receptors, along with another ligand, 2-arachidonyl glycerol [5]. Two G protein-coupled receptors, GPR119 and GPR55, are implicated as membrane surface receptors for OEA and PEA, respectively [6,7]. In the present review, GPR119 and GPR55 are discussed along with the proposed ligands, OEA and PEA, from the perspective of the pharmacology of intercellular lipid mediators and drug discovery.

## 2. OEA and GPR119

### 2.1. Physiological Actions of OEA

#### 2.1.1. Anorectic Action of OEA

OEA was initially described as a lipid that induces anorexia and body weight loss via altering peripheral signals in rodent models [8,9,10]. Currently, OEA is known to be generated from hydrolysis of dietary lipids in the intestinal lumen [8,9,10]. OEA suppresses appetite via various mechanisms, and thus, reduces food intake. Indeed, several targets, such as transient receptor potential vanilloid 1 (TRPV1), peroxisome proliferator-activated receptor-α (PPARα), and GPR119, have been investigated for the anorectic action of OEA.

Further, OEA delays meal initiation and causes reduction in meal size and meal frequency [11]. However, it did not influence food intake when injected into cerebral ventricles; its anorexic action was inhibited by blockage of peripheral sensory fibers upon treatment with capsaicin, implying peripheral regulation on feeding [8]. Food intake stimulates mucosal cells in the duodenum and jejunum to generate OEA. Localized OEA production through food intake in the small intestine of rodents is sufficient to induce a satiety state similar to that induced by systemic OEA treatment [12,13], suggesting that OEA serves as a local messenger for satiety that excites peripheral vagal sensory nerves.

OEA also enhances the magnitude of currents evoked by TRPV1 [14,15]. OEA was reported to induce increase in Ca^2+^ concentrations in capsaicin-sensitive sensory neurons, which were inhibited by capsazepine, a TRPV1 blocker [14,15]. Therefore, TRPV1 was proposed as a potential target of OEA and might be responsible for an excitatory action of OEA on sensory nerves, resulting in satiety response [14,15].

OEA also showed high affinity to PPARα, a nuclear receptor that regulates lipid metabolism. Its administration also induced satiety and body weight loss in wild-type mice but not in PPARα knockout mice, suggesting its role in PPARα activation [16,17].

Weight loss has also been observed after in vivo administration of several GPR119 agonists, such as AR231453, PSN632408, and YH18421 [18,19,20,21]. However, the anorectic action of OEA seems not to be mediated through GPR119 because intraperitoneal injection of OEA suppressed food intake in both wild-type and GPR119 knockout mice; moreover, off-target effects were proposed for AR231453 and PSN632408 [21,22,23]. Recently, OEA was also reported to suppress food intake via activating central oxytocin transmission [24].

**Figure 1:**
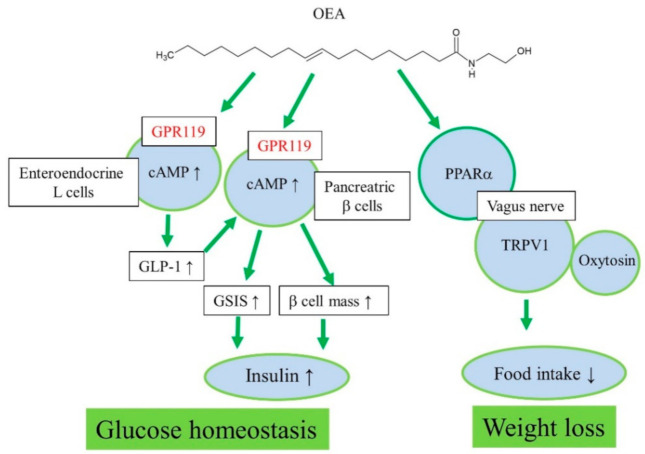
Proposed action mechanisms of OEA for regulation of glucose homeostasis and weight loss.

Therefore, endogenous formation of OEA and other acylethanolamines in the small intestine may play an important role in food intake regulation through PPARα signaling and vagus nerve stimulation to the appetite center in the brain (Figure 1). A chronic high-fat diet causes a decrease in the endogenous levels of anorectic OEA in the intestine and increase in food intake or a hyperphagic effect [13,25].

#### 2.1.2. Analgesic and Other Actions of OEA

OEA showed analgesic action in an experimental animal model; it reduced nociceptive responses that were generated by administration of acetic acid and formalin [26]. It has also been reported to suppress inflammatory and visceral responses through a PPARα-activation independent mechanism [26]. Further, administration of sub-analgesic doses of MK-801 (*N*-methyl-D-aspartate receptor antagonist) and OEA together induced an analgesic effect, implying the contribution of glutamatergic transmission in the antinociceptive action of OEA [26].

Luminal or blood-borne GPR119 agonism by OEA and another agonist, 2-oleoyl-glycerol, can stimulate peptide tyrosine-tyrosine (PYY) release from enteroendocrine L-cells; subsequent paracrine PYY signaling through the Y1 receptor retards colonic motility [27]. The modulation of colonic motility by GPR119 activation was not observed in GPR119 knockout tissue [27]. Plasma levels of OEA and AEA were increased in patients with Crohn’s disease and ulcerative colitis, and levels of lysophosphatidylinositols 18:0 and 20:4 were higher in patients with inflammatory bowel disease than in control subjects. In addition, levels of GPR119 and CB_1_ transcripts were decreased in patients with Crohn’s disease [28], implying the significance of OEA and GPR119 in inflammatory bowel diseases.

OEA treatment has also been shown to increase the abundance of *Akkermansia muciniphila*, a mucin-degrading bacterium in the human intestine having an anti-inflammatory effect, suggesting its use in the treatment of obesity [29]. OEA inhibits osteoclast resorptive function by disrupting cytoskeletal organization, not related to RANKL-induced osteoclast differentiation [30]. It also enhances the differentiation of SZ95 human sebocytes, resulting in increased lipogenesis, promotion of granulated tissue formation, and induction of early apoptotic events; furthermore, OEA shifted the cells to a pro-inflammatory phenotype, resulting in increased production of pro-inflammatory cytokines by these cells [31]. GPR119 expression was downregulated in the sebaceous glands of patients with acne, implying OEA/GPR119 signaling as a positive regulator of sebocyte differentiation [31]. Therefore, OEA and GPR119 have been implicated in analgesia, colon motility and colitis, regulation of microbiota, and bone and skin biology.

### 2.2. Pharmacology of GPR119

#### 2.2.1. GPR119 Expression

Initially, GPR119 was cloned as an orphan GPCR [32] and its expression was found to be predominantly high in insulin-secreting β-cell and GLP-1-producing L-cells, present in the pancreas and the gastrointestinal tract, respectively [21,33,34,35,36]. Additionally, GPR119 mRNA was highly expressed in GIP-producing K-cells and CCK-producing I-cells derived from murine small intestinal cells [37,38,39]; its expression was also reported in rodent brain and human fatal liver [40]. In addition, pancreatic polypeptide (PP)-producing cells and glucagon-producing islet α-cells were suggested to express GPR119 [36,41,42].

#### 2.2.2. GPR119 Ligands

In 2002, Bonini et al. observed constitutive activation of Gαs proteins in GPR119-expressing cells [40], which was consistently observed in later studies as well [21]. In 2005, Soga et al. first reported oleoyl-lysophosphatidylcholine as a ligand for GPR119, along with other lysolipids [33]. In 2006, Overton et al. reported OEA as a ligand for GPR119, along with other acylethanolamides [18]. Later, *N*-oleoyldopamine and 5-hydroxy-eicosapentaenoic acid were also considered as endogenous GPR119 agonists [43,44]. The most potent natural ligands reported were OEA and *N*-oleoyldopamine [18,43]. Therefore, the derivatives of oleic acid showed similar low micromolar potency and full efficacy at GPR119 receptors [18,43]. 2-Oleoyl-glycerol has also been proposed as an endogenous GPR119 ligand, formed during digestion of fats, which activates L-cells to produce GLP-1 in humans [45,46].

#### 2.2.3. GPR119 Functions

GPR119 expression in pancreatic β-cells and intestinal L-cells makes it as an attractive therapeutic target in type 2 diabetes; stimulation of glucose-dependent insulin secretion from β-cell by increased plasma GLP-1 released by L-cells provides new avenues for diabetes treatment using GLP-1 analogs and dipeptidyl peptidase 4 inhibitors.

##### Pancreas

Initially, oleoyl-lysophosphatidylcholine was found to induce insulin secretion from a mouse pancreatic β-cell line, NIT-1, in a GPR119-dependent manner [33]. Oleoyl-lysophosphatidylcholine and OEA increased cAMP and insulin secretion in MIN6c4 and RINm5f insulinoma cells (Figure 1) [23]. Chu et al. reported that activation of GPR119 by AR231453, a specific GPR119 agonist, improved glucose homeostasis via cAMP-mediated enhancement of glucose-dependent insulin release in both rodent islets and the hamster β-cell line, HIT-T15 cells [21]. Further, AR231453 promoted glucose-dependent insulin release in vivo and improved oral glucose tolerance in wild-type mice, but not in GPR119 knockout mice [21]. *N*-Oleoyldopamine induced cAMP accumulation in GPR119-transfected cells as effectively as OEA [43]. Oral administration of *N*-oleoyldopamine to C57bl/6 mice induced significant improvement in glucose tolerance, whereas GPR119 knockout mice were not responsive [43].

Further, GPR119 mRNA levels were elevated in islets of obese hyperglycemic db/db mice compared with those in control islets, implying a possible involvement of GPR119 in the development of diabetes and obesity [41]. In addition, maintaining β-cell mass is very important for glucose homeostasis in diabetic patients. OEA and PSN632408 could stimulate the replication of β-cells in vitro and in vivo, improving islet graft function. GPR119 is a novel therapeutic target for increasing β-cells mass and improving islet graft function via enhancing β-cell replication [20]. However, the cytoprotective actions of OEA in BRIN-BD11 or INS-1E cells do not require GPR119 activation; rather, internalized and hydrolyzed free oleate mediate the cytoprotection [47].

##### GI Tract

GPR119 mRNA was detected in all L-cell models, including murine GLUTag, human NCI-H716, and primary fetal rat intestinal L-cells [35]. OEA increased GLP-1 secretion from intestinal L-cells through GPR119 activation in vitro and in vivo (Figure 1) [35]. In GLUTag-Fla cells, AR231453 induced cAMP accumulation and GLP-1 release [34]. Moreover, AR231453 increased active GLP-1 levels within 2 min after oral glucose delivery in mice, and substantially enhanced the levels of glucose-dependent insulinotropic peptide, but no such induction was observed in GPR119 knockout mice [34]. Nutrient-stimulated GLP-1 release was attenuated in GPR119 knockout mice [22]. Thus, GPR119 regulates glucose tolerance by acting on both intestinal L-cells and pancreatic β-cells [34]. 2-Oleoyl-glycerol activated GPR119 and caused an increase in GLP-1 in the human intestine [45]. Similar results have been observed with other GPR119 agonists in vitro and in vivo [18,48,49,50,51]. AR231453 also induced an increase in GPR119-activation dependent plasma GIP levels [34]. In various animal models of obesity and type 2 diabetes, orally available, potent, selective, synthetic GPR119 agonists lowered blood glucose without hypoglycemia, slowed diabetes progression, and reduced food intake and body weight [46,52,53,54,55,56,57].

### 2.3. Drug Development

Restricted expression and distribution of GPR119 in pancreatic β-cell and intestinal L-cells have drawn great attention in the pharmaceutical industry owing to its potential role in glucose homeostasis. Many drug candidates have been developed based on the dual mechanism of action of GPR119—direct insulin secretion from β-cells and indirect secretion through GLP-1 activation, respectively. In addition, GPR119 agonists with unique scaffolds and signaling properties have been developed [18,48,49,50,51,53,54,55,56,57]. As mentioned above, GPR119 is coupled to Gαs proteins in the gut and pancreas and activated to induce release of GLP-1 and GIP from L-cells and K-cells and of insulin from β-cells. However, basal constitutive activity of GPR119 has been observed, and OEA has demonstrated differential modulation of GPR119-mediated signaling; the potency profiles of OEA with regard to Gαq, Gαi, β-arrestin recruitment, and downstream cAMP response element and serum response element are variable [58]. Many GPR119 agonists, such as GSK1292263, APD668, APD5997, MBX-2982, BMS-903452, LEZ763, ZYG-19, and PSN821, have been developed for type 2 diabetes and obesity [53,54,55,56,57]. However, several clinical trials have failed because repeated administration did not reduce the mean glucose levels in these trials [56,58,59,60].

Recently, continuing efforts on new GPR119 agonist development has resulted in novel structures with better potency and efficacy [19,48,61,62,63]. In particular, DS-8500a has shown positive clinical outcomes for 12 weeks in Japanese patients with type 2 diabetes; the outcomes included HbA1c lowering, glucose lowering, and lipid profile improvement [63]. The sustained efficacy of DS-8500a on glucose control for 12 weeks has revealed an opportunity to overshadow previously failed clinical trials of JNJ-38431055 and GSK1292263 [56,59]. DS-8500a administration also showed improvement in lipid profile, which has been supported by previous preclinical and clinical studies of GSK1292263, PSN821, and JTP-109192 [50,64,65]. These results were also consistent with the results of OEA treatment significantly decreasing plasma triglyceride levels [66].

In addition to the hypophagic effect of OEA, AR231453 induced a reduction in feeding behavior, but only at higher doses than those for glucose homeostasis [21]; also, the hypophagic effects did not disappear in GPR119 knockout mice [34]. In rat models, administration of PSN632408, a synthetic GPR119 agonist, suppressed food intake and reduced body weight gain and white adipose tissue deposition [18], but through GPR119-independent pathways [23]. These results exclude the role of GPR119 as a therapeutic target for the hypophagic action of OEA. However, plasma levels of PYY, an anorexigenic peptide, were increased significantly in patients treated with GSK1292263 and DS-8500a [56,63]. Furthermore, GPR119 expression in PP-cells was reported. GPR119 agonists might have stimulated PP secretion and induced satiety in patients with type 2 diabetes and obesity [41]. Recently, GPR119 activation in pancreatic α-cells was shown to increase glucagon secretion during insulin-induced hypoglycemia [36,42]. In addition, application of GPR119 agonists in non-alcoholic steatohepatitis has been suggested [67]. Further research is needed to confirm the role of GPR119 activation in PYY increase, glucagon secretion, and non-alcoholic fatty liver.

## 3. PEA and GPR55

### 3.1. Pharmacological Actions of PEA

Pharmacological actions of PEA have been investigated in three categories of analgesia, anti-anaphylaxis, and anti-inflammation. The first anti-inflammatory activity of PEA was described by Ganley and Robinson in 1958 [68] and the authors also reported anti-anaphylactic and anti-serotonin activities of PEA obtained from egg yolk, peanut oil, and soybean lecithin [69]. Later, exogenous PEA was reported to exert anti-inflammatory action in a chronic inflammatory disease, Freund’s adjuvant-induced arthritis, in 1971 [70].

#### 3.1.1. Implications of PEA in Anti-Anaphylactic Activity

Aloe et al. reported the pharmacological effects of PEA on mast cells in 1993 [71]. In the study, PEA, when systemically administrated, reduced mast cell degranulation induced by local injection of substance P in the ear pinna of rats [71]. Subsequently, PEA was reported to suppress IgE-triggered activation of mast cells, which was suggested to be mediated by agonism on peripheral CB_2_ receptors in the mast cells. This has been evidenced by suppression of binding of [^3^H]WIN 55212-2b, the full agonist of CB_1_/CB_2_ receptors, by PEA in mast cells expressing CB_2_ only [72,73]. PEA agonist activity on CB_2_ receptor was also observed in cerebellar granule cells in glutamate-induced neurotoxicity [74]. In addition, PEA was found to accumulate in inflamed tissues after injury [75]. Later, the in vivo anti-anaphylactic action of PEA was shown to be mediated by downregulation of serotonin release from basophils and mast cells [76]. In addition, PEA reduced plasma extravasation induced by passive cutaneous anaphylaxis reaction in a dose-dependent manner [76].

Therefore, the acronym ALIA (autacoid local inflammation antagonism) was proposed for the mechanism of action of PEA in the early 1990s [71]. The activation of mast cells led to the production of endogenous PEA, which in turn acted as a local autocrine or paracrine regulator for the negative feedback mechanism [71]. PEA supplementation prevented ovalbumin-induced bronchial hyperreactivity, but it did not affect plasma IgE level [77]. In addition, PEA could act as an entourage compound for endocannabinoids by inhibiting the inactivation of endogenous cannabinoids, thereby increasing their levels [78]. PEA counteracted substance P-stimulated degranulation and histamine release in RBL-2H3 cells, in a manner antagonized by AM630, a CB_2_ antagonist, and by OMDM188, a diacylglycerol lipase enzyme inhibitor (DAGL) [79]. PEA significantly stimulated DAGL-α and -β activity and, consequently, increased biosynthesis of 2-arachidonyl glycerol, which might have contributed to the PEA action [79]. Additionally, PEA levels were significantly reduced in the bronchi of ovalbumin-treated animals, and upregulation of CB_2_ and GPR55 receptors was observed [77].

**Figure 2:**
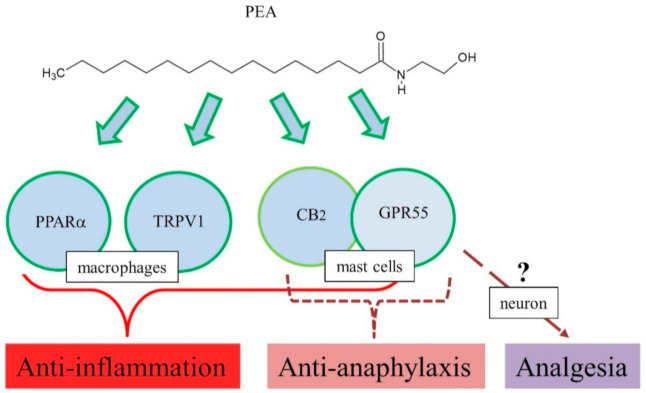
Proposed action mechanisms of PEA for anti-inflammation, anti-anaphylaxis, and analgesia.

Therefore, PEA may negatively regulate anaphylactic responses, such as mast cell degranulation, and its mechanism of action might be related to the endocannabinoid-like system, including CB_2_, GPR55, and 2-arachidonyl glycerol (Figure 2).

#### 3.1.2. Implications of PEA in Anti-Inflammatory Effects

Oral administration of PEA reduced carrageenan-induced hind paw edema in a time- and dose-dependent manner, and significantly decreased carrageenan-induced mechanical hyperalgesia [76]. The anti-inflammatory properties of PEA were shared by the endogenous CB_2_ receptor ligands [78,80]. However, CB_2_ involvement could not be observed in the later study [80].

The anti-inflammatory action of PEA in a rat model of carrageenan-induced acute hind paw inflammation was compared with that of the non-steroidal anti-inflammatory drug, indomethacin [81]. PEA and indomethacin reduced the inflammatory parameters in a time-dependent manner, and the selective CB_2_ antagonist (SR 144528) prevented the anti-edema and anti-hyperalgesic effects of PEA, suggesting its interaction with a yet uncharacterized CB_2_-like cannabinoid receptor [81].

Consistently, the above-mentioned results with CB_2_ agonists and antagonists suggest involvement of CB_2_ receptor in pharmacological actions of PEA [72,81]. However, studies reported no interaction of PEA with CB_1_ and CB_2_ [82,83]. Although CB_2_ involvement is controversial, multiple targets have been suggested for PEA anti-inflammatory actions, GPR55, TRPV1, and PPARα [80]. Treatment with INF-γ and TNF-α increased phosphoprotein and cytokine levels in colonic explants, while PEA significantly reduced the levels of phosphoprotein and cytokine production in colonic explants [84]. In addition, these effects of PEA were blocked by the PPARα antagonist GW6471 [84]. Furthermore, in vitro, PEA decreased inflammation-induced flux of dextrans, sensitive to PPARα [84]. PEA prevented an inflammation-induced increase in PPARα transcription and a decrease in TRPV1 [84]. Immunoreactivity of GPR55 has been observed in the lamina propria macrophages and smooth muscle cells [85]. Mouse primary macrophages were polarized toward pro-inflammatory phenotypes, and reduced *N*-acyl phosphatidylethanolamine phospholipase D expression and PEA bioavailability, and PEA exerted potent anti-inflammatory actions [86], implying the role of GPR55 as a target for PEA action. In addition, PEA has high affinity for PPARα, which mediate many biological effects, including anti-inflammation [3]. In a DNBS-induced colitis model, exogenous PEA administration attenuated inflammation and intestinal permeability and stimulated colonic cell proliferation [87]. This led to increased colonic levels of PEA and endocannabinoids, downregulation of GPR55 mRNA, but no changes in CB_1_, CB_2_, and PPARα [87]. Therefore, PEA exerted anti-inflammatory responses in several animal models, and its mechanism of action might be related to several targets such as PPARα, TRPV1, GPR55, and CB_2_ (Figure 2) [80].

#### 3.1.3. Implications of PEA in Analgesic and Neurologic Activities

PEA showed low affinity for CB_1_ and CB_2_ receptors, yet selectively activated GPR55 receptors [3]. A possible role of GPR55 receptors in the anti-nociceptive activity of PEA has been implicated in formalin-induced pain-related behavioral modifications [88]. Furthermore, PEA actions in social interaction and neural transmission have been reported as follows.

PEA-induced activation of GPR55 in the ventral hippocampus, a critical region for cognition, recognition memory, and affective processing produced a hyperdopaminergic state in the mesolimbic dopaminergic system, which means an increased firing and bursting activity of dopaminergic neuron populations of the ventral tegmental area [89]. This resulted in strong disruptions in context-independent associative fear memory formation, social interaction, recognition memory, and spatial location memory [89].

Prenatal exposure to valproic acid in rats reduced GPR55 expression in the frontal cortex and hippocampus. In addition, levels of acylethanolamides, such as PEA, OEA, and AEA, were higher in the hippocampus of these rats immediately following social exposure, implying the involvement of GPR55 and its agonist acylethanolamides in social interaction [90].

PEA was found to enhance GABA transmission and triggered the synthesis of 2-arachidonyl glycerol as well, at the postsynaptic site in the striatum, which in turn inhibited GABA release in a retrograde manner through the stimulation of presynaptic CB_1_ receptors, implying complex neural networks between GABA neurons and PEA dynamics [91].

PF3845 is an inhibitor of FAAH, an acylethanolamide catabolic enzyme. Systemic administration of PF3845 increased the levels of PEA, OEA, and AEA in the hippocampus and frontal cortex of rats in vivo in TLR4-induced neuroinflammatory responses [92], implying dynamic turnover of PEA in the brain along with other acylethanolamides.

There are many reports on the production of PEA and other acylethanolamides and expression of GPR55 in the brain. These reports indicate the functions of PEA and GPR55 in analgesia, social interaction, and neural transmission.

#### 3.1.4. Other Effects

PEA caused a concentration-dependent increase in outflow facility in an anterior segment porcine organ culture model, which was speculated to be mediated by GPR55 and PPARα [93]. PEA also inhibits vasopressor responses to exogenous noradrenaline or sympathetic stimulation, which induces hypotension. The PEA action is reported to be mediated by prejunctional and vascular CB_1_, TRPV1, and probably GPR55 [94].

### 3.2. Pharmacology of GPR55

Ryberg et al. reported GPR55 as a novel cannabinoid receptor because cannabinoid ligand CP55940 exhibits specific binding to GPR55 [6]. PEA is one of the proposed ligands including cannabidiol and abnormal cannabidiol. EC_50_ values of acylethanolamides, such as PEA, AEA, OEA, and 2-arachidonyl glycerol, were measured as 4, 18, 440, and 3 nM in GTPγS binding, respectively [6]. However, in other assay systems, PEA did not exhibit a statistically significant effect in GPR55-transfected cells [95,96,97]. The most potent endogenous lipid for GPR55 known is 2-arachidonyl lysophosphatidylinositol [96,97,98,99]. Phylogenetically, GPR55 belongs to a cluster of lipid receptors (GPR35, LPA_4_/P2Y9, LPA_5_/GPR92, LPA_6_/P2Y5, FFA1/GPR40, FFA2/GPR43, and FFA3/GPR41) [6].

The mechanism of action of PEA has been studied, and many molecular targets have been proposed, including CB_2_, GPR55, TRPV1, and PPARα [78]. The anti-anaphylactic, anti-inflammatory, and analgesic effects of PEA were reversed by CB_2_ antagonists [72,78,81], thus implicating CB_2_ receptor as a target of PEA. However, another putative CB-like receptor was present in the RBL-2H3 cells [78]. GPR55 was identified as a receptor that could be implicated in mast cells [6]. However, there has been no further report on this subject. Another possible mechanism suggested that the effects of PEA involve indirect regulation of microglial CB_2_ expression [100]. PEA was found to increase the mRNA and protein expression of CB_2_ through PPARα activation [100]. In addition, crosstalk between GPR55 and the cannabinoid receptors (CB_1_ and CB_2_) via heteromerization is intriguing. In the co-expression system, CB_1_ signaling was enhanced by GPR55 expression, and cross-antagonism between GPR55 and CB_2_ was observed [101,102]. Further research on the mechanism of action of PEA in mast cells via the GPR55-CB_2_ complex is needed.

Although GPR55 is reported to be a receptor for lipid mediators such as lysophosphatidylinositol and PEA, there have not been many studies on GPR55 being a PEA receptor. Instead, many studies have been conducted using GPR55 knockout mice and the most potent natural ligand, 2-arachidonyl lysophosphatidylinositol, or a synthetic agonist, O-1602 [103,104,105]. GPR55 is ubiquitously expressed, and its implications in anxiety, bone development, cancer, inflammation, metabolic disturbance, nociception, and synaptic transmission have been studied [6,104]. In pain, GPR55 knockout abolished mechanical hyperalgesia following inflammatory or neuropathic insults, but this was not observed in a later study [105,106]. In experimental colitis model, GPR55 knockout mice showed less severe symptoms, and CID16020046, a potent GPR55 antagonist, reduced colon inflammation [107,108]. Inhibitory effects of O-1602 on intestinal contractility and colonic motility is a GPR55-dependent process [109,110]. The role of GPR55 in memory processing, social interaction, and synaptic transmission has also been reported [92,111,112]. However, there are inconsistencies in the reports regarding the role of GPR55 given findings related to the phenotypic characterization of GPR55 knockout mice and GPR55-independent actions of O-1602 and CID1602046 [103,105,106,107,113,114,115]. So far, increasing evidence supports lysophosphatidylinositol instead of PEA as an endogenous ligand for GPR55 [6,95,96,97]. Published information on GPR55 functions is complicated by different phenotypic characterization of GPR55 knockout mice and off-target effects of O-1602 and CID1602046 [103,105,106,107,113,114,115]. Therefore, it is crucial to identify specific and selective agonists/antagonists for GPR55 in future research.

## 4. Concluding Remarks

Treatment with acylethanolamides OEA and PEA elicit multiple responses through multiple targets. The anti-inflammatory action of PEA may be mediated partially through PPARα. The anti-anaphylactic action of PEA was studied in the early 1990s, and involvement of CB_2_ or CB_2_-like receptors has been implicated. However, the likelihood of GPR55 as CB_2_-like receptor and heterodimeric functions of CB_2_ and GPR55 in the mast cells have not been studied. Despite being an endogenous ligand for GPR55, the pathophysiological significance of lysophosphatidylinositol in association with GPR55 has poorly been studied in detail. Instead, substantial research work focused on synthetic agonist O-1602 and antagonist CID16020046 in GPR55 knockout mice, which resulted in complex and contradictory reports. Thus, many selective and specific agonists/antagonists need to be developed with respect to GPR55 receptors.

The anorectic action of OEA seemed to be mediated mainly through PPARα and peripheral vagal sensory nerves. Since the discovery of GPR119 as a receptor for OEA, development of GPR119 agonists as potential pharmacotherapies for diabetes and obesity has been intensively conducted. OEA-induced glucose homeostasis can be mediated by GPR119 receptor, which influences GLP-1 release from L-cells and insulin secretion from β-cells. Drug development targeting GPR119 has been boosted by novel scaffolds such as DS-8500a. However, several synthetic GPR119 agonists induce body weight loss by a GPR119-independent process. In addition, little is known about GPR119 functions in pancreatic α-cells and PP-cells and in PYY secretion from L-cells. Further research is needed in a variety of cell types and organs to elucidate the pathophysiological role of GPR119 in type 2 diabetes and obesity.

## Data Availability

Not applicable.

## Abbreviations

OEA	Oleoylethanolamide
PEA	Palmitoylethanolmide
PPARα	Peroxisome proliferator-activated receptor-α
TRPV1	Transient receptor potential vanilloid 1
DAGL	Diacylglycerol lipase
